# Nanoparticles to Enhance Melting Performance of Phase Change Materials for Thermal Energy Storage

**DOI:** 10.3390/nano12111864

**Published:** 2022-05-30

**Authors:** Yu Han, Yan Yang, Tapas Mallick, Chuang Wen

**Affiliations:** 1 School of Mechanical and Electrical Engineering, Suqian University, Suqian 223800, China; yuhan.neu@gmail.com; 2 School of Mechanical Engineering and Automation, Northeastern University, Shenyang 110819, China; 3 School of Petroleum Engineering, Changzhou University, Changzhou 213164, China; 4 College of Engineering, Mathematics and Physical Sciences, University of Exeter, North Park Road, Exeter EX4 4QF, UK; t.k.mallick@exeter.ac.uk

**Keywords:** phase change material, energy storage, nanoparticles, nanofluid, melting process, PCM, heat transfer, natural convection, liquid-solid interface

## Abstract

The present study proposes the phase change material (PCM) as a thermal energy storage unit to ensure the stability and flexibility of solar-energy-based heating and cooling systems. A mathematical model is developed to evaluate the PCM melting process, considering the effect of nanoparticles on heat transfer. We evaluate the role of nanoparticles (Al_2_O_3_-, copper- and graphene-based nanofluids) in enhancing the performance of the melting process of phase change materials. The results show that natural convection due to the buoyancy effect dominates the flow behaviour even in the initial stage of the PCM melting process. High natural convection at the bottom of the annular tube moves the melted PCM upward from the lateral, which pushes the liquid–solid interface downward. The addition of 3% vol Al_2_O_3_ nanoparticles boosts PCM melting performance by decreasing the melting time of PCM by approximately 15%. The comparison of Al_2_O_3_, copper and graphene nanoparticles demonstrates that higher thermal conductivity, ranging from 36 to 5000 W m^−1^ K^−1^, does not contribute to a significant improvement in the melting performance of PCMs.

## 1. Introduction

Solar and wind power are renewable and sustainable energy sources that have received extensive attention. Current trends show the growth of global demand for renewable energy sources. Thermal energy storage (TES) is considered to be an effective way to store renewable energy. This system has various applications, such as solar water heaters and building air-conditioning systems [1,2,3]. There are three options for storing energy in TES systems: manifesting, potentiality, and thermochemistry. These options are more attractive when compared with other options because of the relatively high storage density and the fact that it is almost possible to achieve isothermal storage conditions. Proposed TES systems that use phase change materials (PCMs) [4] can store several times more energy than systems using sensitive storage materials, even when the same material types and volumes are used [5]. However, most phase change materials have the disadvantageous property of relatively low thermal conductivity [6], which strongly inhibits the energy charge and discharge rates. As such, it is necessary to improve system response time to meet the requirements. One way to overcome this problem is by modifying the PCM container structure, such as by using fins [7,8,9], heat pipes [10,11] and metal foam [12,13,14].

Among these enhancement technologies, fins are the most widely used heat transfer technology in engineering applications, including PCM-based TES applications. Metal fins are one of the most practical heat transfer enhancement technologies in the current market due to their high efficiency, ease of manufacture and low cost of construction. Kamkari and Shokouhmand [15] reported on an experimental study on the melting process and the effect of fins on a storage cavity containing PCM. In recent years, various fin shapes have been investigated to increase the performance of fin systems in transmitting heat to the PCM. Sciacovelli et al. [16] studied the effect of adding fins to a shell-and-tube latent heat energy storage system (LHESS). Computational fluid dynamics (CFD) are used to optimize the geometry of tree fins with one and two forks. The results of the optimized configuration showed an approximately 24% increase in heat transfer efficiency. Gharebaghi and Sezai [17] performed a numerical study on a finned heat sink, filled with RT27 as its PCM, and performed analyses on horizontal and vertical arrangements. The results indicated that the heat transfer rate could be increased up to 80 times by increasing the number of fins for higher temperature differences and speeding up the melting process by reducing the fin spacing. Rathod and Banerjee [18] presented a practical study on paraffin melting and solidification as a PCM. The experiment investigated the effect of PCM in a vertical shell-and-tube heat exchanger assisted by longitudinal fins. After the installation of fins, the results showed that the total time required for the PCM to melt was reduced by 25%, and the total time required to solidify was reduced by 44%. The study results from Agyenim et al. [19] also indicated that longitudinal fins showed the best performance for heat transfer applications, with reduced subcooling during the discharge.

The heat transmission efficiency of PCM can also be enhanced with the addition of highly conductive nanoparticles [20,21]. Khodadadi and Hosseinizadeh [22] introduced the concept of combining nanoparticles with PCM to enhance the thermal response. The numerical simulation showed that adequate phase change heat transfer could be achieved by introducing nanoparticles in TES applications. Sciacovelli et al. [23] studied the thermal behaviour of potential TES units equipped with nano-enhanced PCM. The melting time was reportedly reduced by 15% by doping nano-enhanced PCM. Lin and Al-Kayiem [24] claimed that the dispersion of copper nanoparticles in paraffin could improve its thermal conductivity and thermal stability as well as reduce the supercooling effect in the discharge stage. Mahdi and Nsofor [25] studied the effect of different sizes of fins and different volume fractions of nanoparticles on the evolution of the melting process. This study observed the solid–liquid interface, isotherm distribution and time distribution of the liquid fraction. The results showed that using fins alone yielded better performance than using either nanoparticles alone or a combination of fins and nanoparticles. Longer fins with decreased thickness are recommended to improve the phase change heat transfer and minimize the volume occupied in the energy storage space. Another study conducted by the same authors [26] used specially arranged fins to achieve an increase in the melting rate of PCM. The results showed that the use of long fins in the lower part of the storage cell, where conduction predominates, results in rapid melting. The studies further discovered that using a minimum number of relatively short fins in the upper half of the cell resulted in better performance. These studies indicate that the successful dispersion of nanoparticles could result in higher thermal conductivity and better PCM heat storage.

The objective of this study ito determine the role of nanoparticles in enhancing the performance of the PCM melting process. Based on the aforementioned analysis, the novelty and contents of this study include:

(a) We propose to integrate PCM into a solar-energy-based heating and cooling system as a thermal energy storage unit, which is expected to ensure the stability and flexibility of the system and mitigate the carbon emissions from the built environment, helping to achieve a net-zero society. The melting process of PCM in a tubular unit is described in detail, considering natural convection due to the buoyancy effect.
(b) The performance of nanoparticles during the melting process of phase change materials is analysed in a tubular energy storage unit. We use an experimentally validated formula for the thermal conductivity of nano-PCM. Factors considered in this study include those usually neglected in previous studies, such as the effects of the Brownian motion of nanoparticles, size, volume fraction and temperature dependence [27,28,29].
(c) We determine the mechanism of performance enhancement of the melting process of PCM using nanoparticles with various thermal conductivities, based on the analysis of Al_2_O_3_-, copper- and graphene-based nanofluids.

## 2. Problem Statements

### 2.1. Phase Change Material Thermal Energy Storage System

**Figure 1:**
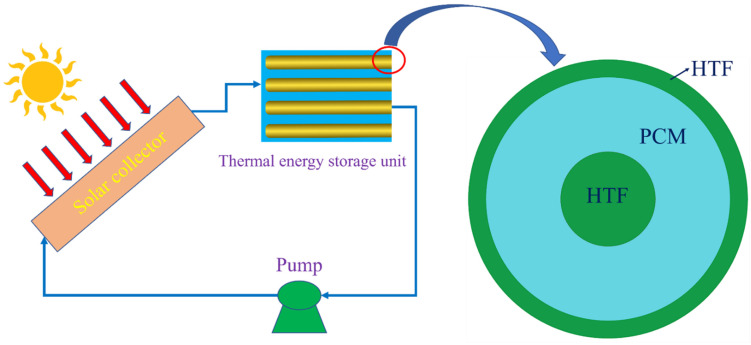
Solar energy heating system with a thermal energy storage unit integrated with phase change materials.

The present study proposes a solar collector system integrating a thermal energy storage unit containing phase change materials. This system mitigates the carbon emissions from the built environment, helping to achieve a net-zero society. Apart from that, the utilization of solar energy in the cooling and heating systems of buildings reduces reliance on natural gas, which is currently the main fuel used in the UK’s residential building heating systems. In addition, the integration of an energy storage system ensures the stability and flexibility of renewable-energy-based heating and cooling systems because it mitigates the intermittency of solar energy. Figure 1 shows a solar energy heating system containing a PCM-based thermal energy storage unit, which illustrates the combination of PCM and heat transfer fluid (HTF) in a triple concentric-tube heat exchanger. This study credits the Al_2_O_3_ nanoparticles employed for the enhancement of the PCM melting process that improves the performance of thermal energy storage in the solar heating systems of the built environment.

**Figure 2:**
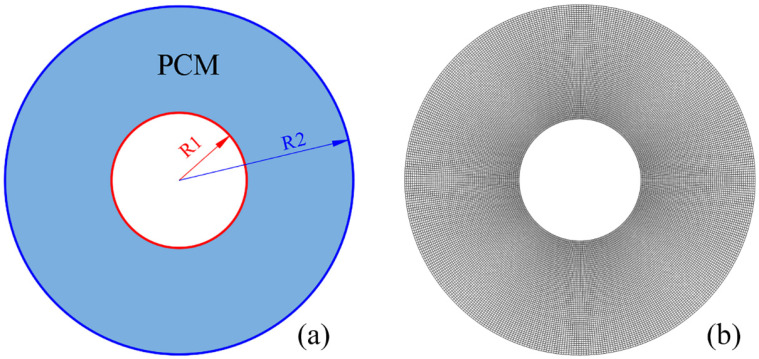
Geometry (**a**) and computational mesh (**b**) for the tubular phase change material thermal energy storage unit.

We focus on the PCM melting process in the numerical simulation; thus, the inner HTF tube and the outer annular tube are not taken into account. Figure 2 illustrates the geometrical model that is used for numerical modelling. The structured mesh is generated for the computational study, as shown in Figure 2b. The radius measurements of the inner and outer tubes are 25.4 mm and 75 mm, respectively.

### 2.2. Governing Equations

Assumptions made for the PCM melting process are that (a) the temperature variation in the HTF is negligible, (b) viscous dissipations are negligible, (c) the Boussinesq approximation is valid, (d) there is no heat loss or gain from the surroundings; (e) there are no-slip conditions for velocities at the boundaries, (f) the volume variation associated with the phase change is negligible. Thus, the equations governing the fluid behaviour are as follows:

(1) ∇·V=0

(2) $$\frac{\partial u}{\partial t}+V\cdot \nabla u=\frac{1}{\rho_{npcm}}\left(-\nabla P+\mu_{npcm}\nabla^{2}u\right)+Cu\frac{{\left(1-\lambda \right)}^{2}}{\lambda^{3}+\varepsilon }$$

(3) $$\begin{array}{c} \frac{\partial v}{\partial t}+V\cdot \nabla \nu =\frac{1}{\rho_{npcm}}(-\nabla Ρ+\mu_{npcm}\nabla^{2}\nu \\ \;\;\;\;\;\;\;\;\;\;+{\left(\rho \beta \right)}_{npcm}g\left(Τ-{Τ}_{ref}\right))+C\nu \frac{{\left(1-\lambda \right)}^{2}}{\lambda^{3}+\varepsilon } \end{array}$$

(4) $$\frac{\partial h}{\partial t}+\frac{\partial \left(\Delta H\right)}{\partial t}+\nabla \cdot \left(Vh\right)=\nabla \cdot \left(\frac{k_{npcm}}{{\left(\rho C_{p}\right)}_{npcm}}\nabla H\right)$$

where ΔH and *h* are latent heat and sensible enthalpy, respectively:

(5) $${h=h}_{ref}+\int_{T_{ref}}^{T}C_{p}dT$$

(6) ΔH=λΓ

where *λ* is the liquid fraction during the phase change:

(7) $$\lambda =\{\begin{array}{cc} 0, & T\leq T_{s}\\ \left({T-T}_{s}\right)/\left(T_{l}{-T}_{s}\right) & {\;T}_{s}{<T<T}_{l}\\ 1, & T\geq T_{l} \end{array}$$

where *T_s_* and *T_l_* are the solidus temperature and liquidus temperature of PCM, respectively.

### 2.3. Thermophysical Properties

The phase change material used in this study is RT82. RT82 consists of organic pure materials that use their melting process from solid to liquid (and vice versa) to store and release large amounts of heat in an approximately constant temperature range. Depending on their melting points, a variety of heat storage applications at different temperatures can be considered. The nanoparticles that are expected to enhance the heat transfer are Al_2_O_3_. The inner and outer walls are copper to achieve a high rate of conduction. The thermophysical properties of RT82, Al_2_O_3_, copper and graphene [30] are listed in Table 1 and Table 2. All of the nanoparticles studied in this work are the same size and shape.

The density, specific heat capacity, latent melting heat and thermal expansion coefficient of the nano-PCM can be examined based on the simple theoretical model of mixing:

(8) $$\rho_{npcm}=\varphi_{n}\rho_{np}+\left(1-\varphi_{n}\right)\rho_{pcm}$$

(9) $${\left(\rho C_{p}\right)}_{npcm}=\varphi_{n}{\left(\rho C_{p}\right)}_{np}+\left(1-\varphi_{n}\right){\left(\rho C_{p}\right)}_{pcm}$$

(10) $${\left(\rho \Gamma \right)}_{npcm}=\left(1-\varphi_{n}\right){\left(\rho \Gamma \right)}_{pcm}$$

(11) $${\left(\rho \beta \right)}_{npcm}=\varphi_{n}{\left(\rho \beta \right)}_{np}+\left(1-\varphi_{n}\right){\left(\rho \beta \right)}_{pcm}$$

where $\varphi_{n}$ is the volume fraction of the nanoparticles, and the subscripts *np*, *npcm* and *pcm* refer to the nanoparticle, nano-PCM and pure base PCM, respectively.

The dynamic viscosity and thermal conductivity of the nano-PCM can be written using the formula experimentally studied by Vajjha et al. [31]:

(12) $$\mu_{npcm}=0.983e^{\left(12.959\varphi \right)}\mu_{pcm}$$

(13) $$k_{npcm}=\frac{k_{np}+2k_{pcm}-2\left(k_{pcm}-k_{np}\right)\varphi_{n}}{k_{np}+2k_{pcm}+\left(k_{pcm}-k_{np}\right)\varphi_{n}}k_{pcm}+5\times 10^{4}\beta_{k}\zeta \varphi_{n}\rho_{pcm}C_{p.pcm}\sqrt{\frac{BT}{\rho_{np}d_{np}}}f\left(T,\varphi_{n}\right)$$

where B is Boltzmann constant, 1.381 × 10^−23^ J/K, $\beta_{k}$ = 8.4407(100*φ_n_*)^−1.07304^, and f is a function defined as:

(14) $$f\left(T\cdot \varphi_{n}\right)=\left(2.8217\times 10^{-2}\varphi_{n}+3.917\times 10^{-3}\right)\frac{T}{T_{ref}}+\left(-3.0669\times 10^{-2}\varphi_{n}-3.91123\times 10^{-3}\right)$$

Formula (13) considers the effect of Brownian motion due to extra nanoparticles that are implemented in the second term of function $f\left(T\cdot \varphi_{n}\right)$.

### 2.4. Numerical Implementations

The numerical simulation is performed using ANSYS Fluent 18.2, while the thermophysical properties are integrated into Fluent using user-defined-function (UDF) interfaces. The numerical simulation is transient because the melting process of PCM is highly time-dependent. The flow behaviour is a laminar flow. The SIMPLE scheme is used for the pressure–velocity coupling while discretizing the pressure by the PRESTO method and the equations of momentum and energy by the second-order upwind method. As this study focuses on a melting process with a transient model, we assume that the initial conditions include a PCM temperature of 300.15 K at the beginning of the computation to ensure that the PCM is in a solid state. For the boundary conditions, both the inner and outer walls are assigned a constant temperature of 363.15 K to heat the PCM. The convergence criteria are below 1.0 × 10^−6^ for all dependent variables.

## 3. Results and Discussion

### 3.1. Model Validation and Verification

#### 3.1.1. Model Sensitivity of Grid Density and Time Step

**Figure 3:**
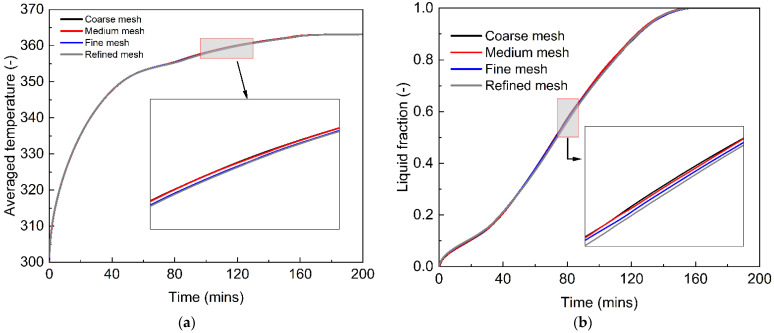
Effect of grid density on the computational results of the melting process of phase change materials. (**a**) Average temperature; (**b**) Liquid fraction.

The grid density is one of the key factors that influence the numerical prediction of the PCM melting process for energy storage purposes. The selection of the grid density is based on the geometry of the computational domain and the numerical cost for the melting process of phase change materials. We employ four different grid densities to test the mesh independence, including a coarse mesh of 13,800 cells, a medium mesh of 25,724 cells, a fine mesh of 45,144 cells and a refined mesh of 94,724 cells. The four computational cases use the same numerical implementations and physical geometries, and the results are shown in Figure 3, including the time, average temperature and liquid fraction of the computational domain. It can be seen that, in our case, the four different grid densities predict almost the same profiles of average temperature and liquid fraction, although slight differences are observed in Figure 3a,b. Considering the computational accuracy and costs, we choose the medium mesh of 25,724 cells for the numerical studies on the PCM melting process in a tubular energy storage system.

**Figure 4:**
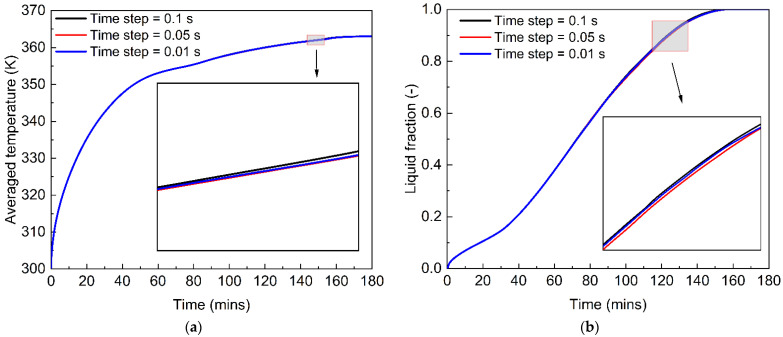
Effect of time step on the computational results of the melting process of phase change materials. (**a**) Average temperature; (**b**) Liquid fraction.

Furthermore, as the PCM melting process is highly time-dependent, the time step is very important for carrying out an unsteady prediction of the phase change behaviour. The time step can affect the accuracy of the numerical studies. With a large time step, the detailed convection heat transfer may not be captured during the PCM melting process. However, while a small time step can improve the accuracy, it also significantly increases the computational cost. Figure 4 describes the average temperature and liquid fraction during the PCM melting process under three time steps of 0.01 s, 0.05 s and 0.1 s. We see that the increase in the time step from 0.01 s to 0.1 s almost does not influence the phase change behaviour of PCM in a tubular energy storage system. Thus, a time step of 0.1 s is employed for the numerical study of the PCM melting process, considering the computational accuracy and costs.

#### 3.1.2. Model Validation against Experimental Data

**Figure 5:**
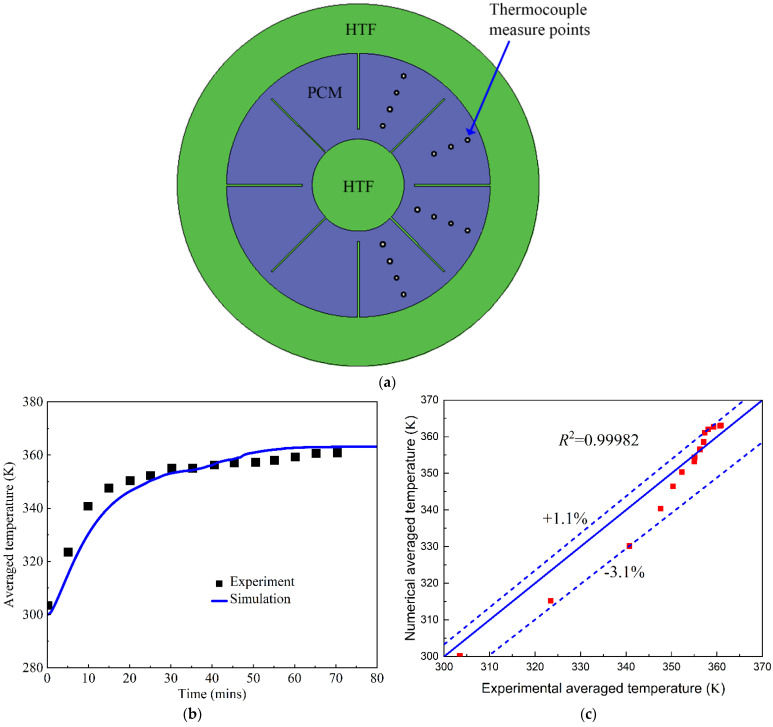
Model validation of the melting process of phase change materials. (**a**) Thermocouple measure points in the experiments [32]; (**b**) Average temperature; (**c**) Error analysis.

Based on the verification of the grid density and time step, the developed CFD model is validated against an experimental test of the melting process performed by Al-Abidi et al. [32]. In their experiments, triplex tube heat exchangers were employed as a latent heat thermal energy storage system integrated with PCM, as shown in Figure 5a. The structured fins in the inner and middle walls were used to enhance the PCM melting process. They measured the average temperature of 15 points during the PCM melting process. We employ the same operating conditions and geometrical parameters for our numerical simulations as a model validation. The root-mean-square (*R*^2^), defined in Equation (7), is used to compare the numerical and experimental errors [33,34,35]. The comparison of the average temperatures in the numerical and experimental data is presented in Figure 5. It can be observed in Figure 5b that the average temperature from the numerical prediction is lower than the experimental measurement in the initial 40 min; the maximum error reaches −3.1% for these cases. After 50 min, the numerical average temperature is higher than the experimental test, with a maximum error of 1.1%. This means that the maximum error for the numerical simulation of the average temperature is no more than ±3.1% for the entire PCM melting process, as shown in Figure 5c. Thus, our developed CFD model is accurate in predicting the PCM melting process for thermal energy storage.

(15) $$R^{2}=1-\frac{\sum_{i=1}^{n}{\left(a_{i}-p_{i}\right)}^{2}}{\sum_{i=1}^{n}{\left(p_{i}\right)}^{2}}$$

where *a_i_* and *p_i_* are experimental and numerical values, respectively.

### 3.2. Melting Process and Interface Evolution of PCM

**Figure 6:**
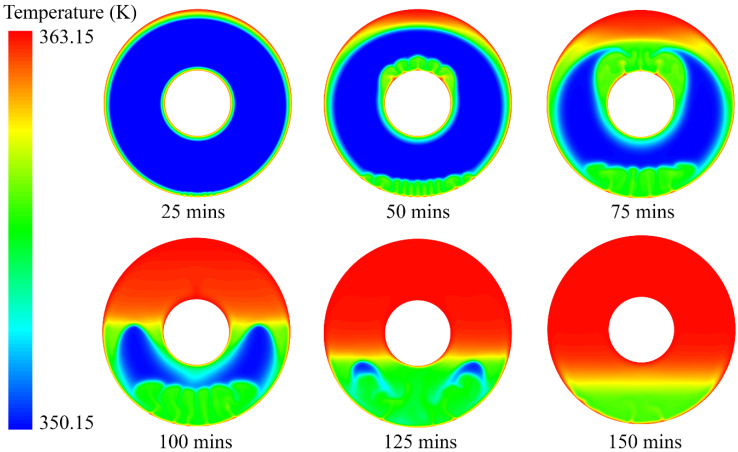
Temperature distribution of PCM during the melting process.

**Figure 7:**
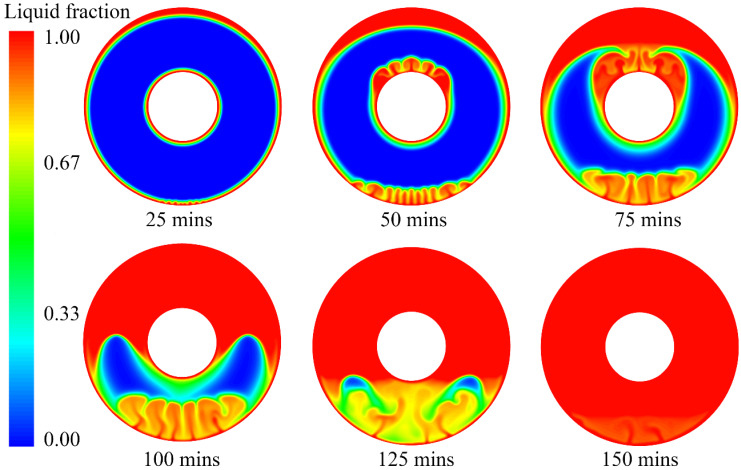
Liquid fraction of PCM during the melting process.

To understand the PCM melting process, the temperature distribution and liquid fraction are employed to describe the phase change behaviour, as shown in Figure 6 and Figure 7. It can be seen from Figure 6 that there is a strong heat transfer flux at the near-wall region when the wall temperature is fixed at 363.15 K. The transition of the colour in the contours in Figure 6 from blue to red indicates a change in temperature from low to high during the melting process. The high level of heat transfer between the wall and PCM results in the natural convection of the melted PCM. For example, even in the initial stage of the PCM melting process (*t* = 25 min), natural convection is observed at the bottom of the annular PCM domain. This indicates that buoyancy dominates the PCM melting process in the annular tube. Furthermore, it can be seen that there are clear liquid–solid interfaces between the melted liquid and solid PCM near the hot inner and outer walls. The thickness of the melted PCM at the top is much higher than that at the bottom of the annular tube. The melted PCM, due to the high heat transfer at the outer wall, presents an eccentric shape at *t* = 25 min. As time goes on, the inner and outer walls heat the PCM near them separately, and the heat transfer accelerates the effect of natural convection due to the buoyancy. It can be observed that the bottom of the outer wall and the top of the inner wall both generate strong natural convections at *t* = 50 min. At that point in the process, the inner and outer walls still heat the PCM near them separately.

The first liquid–solid interface is around the outer wall, which moves to the centre of the annular tube. Two factors contribute to the movement of this interface. One is the increase in melted PCM due to the heating effect of the hot outer wall. The other is the hot melted PCM moving from the bottom due to natural convection. The increase in the interface rate at the top and bottom is larger than that at the lateral, which is demonstrated by the liquid fraction in Figure 7 at *t* = 25 min and *t* = 50 min. Similarly, the second liquid–solid interface is observed around the hot inner wall, and the melted PCM grows thicker during the melting process. It also can be seen that the interface at the top of the inner wall moves faster than those at the bottom and lateral of the inner tube. The movements of the two liquid–solid interfaces indicate that natural convection dominates the PCM melting process.

In the middle stage of the melting process, the melted PCM from the outer wall succeeds in meeting the melted PCM from the inner wall in the upper half at *t* = 75 min. The first and second liquid–solid interfaces merge in the upper half and then start to move downward. The merging of the two interfaces promotes natural convection during the PCM melting process, and more PCM is melted due to strong natural convection, resulting in higher liquid fractions that account for more than 50% at that point in the melting process. However, the two interfaces do not meet each other in the lower half, even though the first liquid–solid interface grows significantly from the bottom of the outer wall. The PCM in the upper half is completely melted at *t* = 100 min, while the melted PCM from the outer wall finally meets the melted PCM from the inner wall in the lower half. The merging of these two liquid–solid interfaces in the lower half dramatically improves natural convection for the PCM melting process.

When the PCM melting process reaches the final stage, the residual solid PCM in the lower half starts to melt due to the high level of natural convection. During this melting process, the melted PCM at the bottom moves upward, which drives the completely melted PCM to move downward. The liquid–solid interface reaches the bottom of the inner tube at *t* = 125 min, but we can still observe tiny amounts of residual solid PCM, and there is strong natural convection in the lower half at this point in the process. Even at *t* = 150 min, we can still observe a very tiny amount of natural convection at the bottom of the annular tube, as shown in Figure 6 and Figure 7. The complete melting time for this case is approximately 155 min.

**Figure 8:**
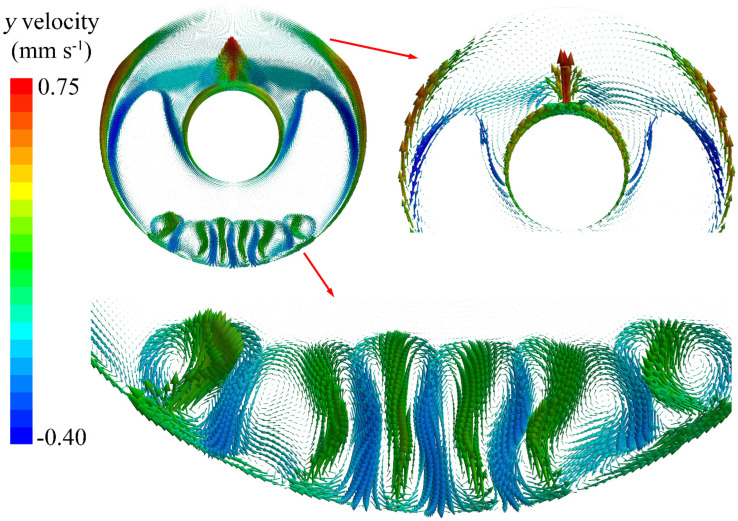
*y* velocity vectors during the PCM melting process at *t* = 85 min.

Figure 8 describes the *y* velocity vectors during the PCM melting process at *t* = 85 min, which exhibits a flow velocity in a vertical direction. It can be seen that natural convection dominates the phase change behaviour during the PCM melting process in the annular tube. For the outer wall, there is strong natural convection at the bottom that generates melted PCM, which moves upward along with the lateral of the outer wall. For the inner wall, the melted PCM gathers at the top of the inner wall due to the natural convection generated by the hot boundary. The combination of the two natural convections from the outer and inner walls leads to the growth of the melted PCM in the upper half; this drives the liquid–solid interface to move downward during the PCM melting process.

### 3.3. Role of Nanoparticles in the PCM Melting Processes

**Figure 9:**
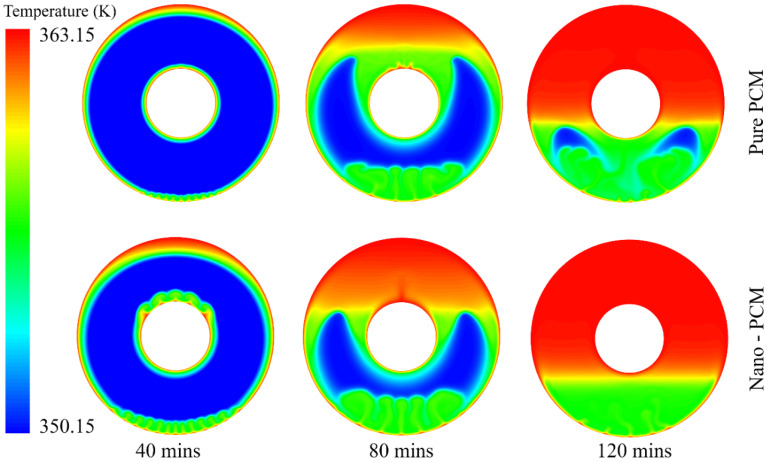
Temperature distribution of PCM during the melting processes with and without nanoparticles.

**Figure 10:**
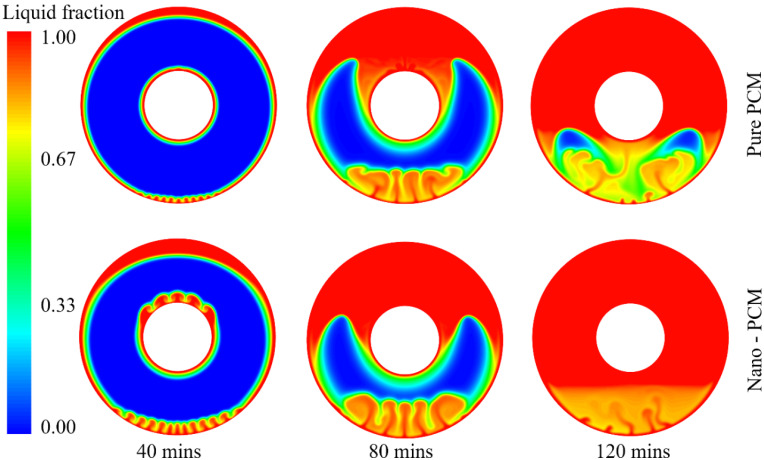
Liquid fraction of PCM during the melting processes with and without nanoparticles.

The PCM is a suitable candidate for energy storage systems due to its high capacity to store latent heat thermal energy. However, one of the disadvantages of PCM is its low thermal conductivity, i.e., 0.20 for RT82 in this study. One of the valid solutions for improving its thermal conductivity is to add nanoparticles with high thermal conductivity into the PCM. In this study, we determined the role of nanoparticles in enhancing the PCM melting process by using 3% vol Al_2_O_3_ nanoparticles. The temperature and liquid fraction are shown in Figure 9 and Figure 10, both with and without nanoparticles, during the PCM melting process.

The addition of Al_2_O_3_ nanoparticles significantly enhances the melting performance of PCM during the initial stage. For example, the thickness of the melted PCM around the hot outer wall with Al_2_O_3_ nanoparticles is approximately twice that of the pure PCM at *t* = 40 min. In addition, the Al_2_O_3_ nanoparticles dramatically improve natural convection at the bottom of the outer wall compared to the pure PCM case. For the inner wall, the added Al_2_O_3_ nanoparticles enhance the natural convection that moves the melting PCM from the bottom to the top. Thus, the top of the inner wall exhibits higher natural convection than in the pure PCM case.

At the middle stage of the PCM melting process, the liquid–solid interface of PCM with Al_2_O_3_ nanoparticles moves further downward than it does for pure PCM. At *t* = 80 min, the liquid–solid interface is above the top of the inner wall for the pure PCM case, while it is below the top of the inner wall for PCM with Al_2_O_3_ nanoparticles. At the final stage of *t* = 120 min, some solid PCM is still observed in the lower half in the pure PCM case, while the minimum liquid fraction is about 0.7 for PCM with Al_2_O_3_ nanoparticles. This demonstrates that including the Al_2_O_3_ nanoparticles in the PCM significantly enhances the PCM melting performance.

**Figure 11:**
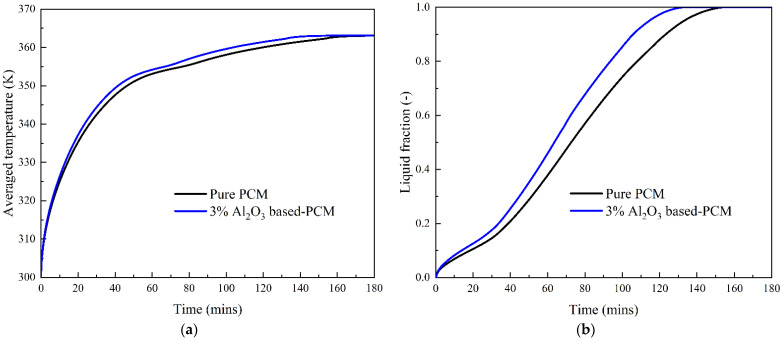
Effect of Al_2_O_3_ nanoparticles on the PCM melting process. (**a**) Average temperature; (**b**) Liquid fraction.

Figure 11 shows the average temperature and liquid fraction during the PCM melting process with and without Al_2_O_3_ nanoparticles. We can see that there is almost no difference in the average temperature of these two cases in the early stage, at 10 min. Along with the progress of the melting process, the average temperature is much higher in PCM with Al_2_O_3_ nanoparticles than it is in the pure PCM case. Adding Al_2_O_3_ nanoparticles into the PCM accelerates the melting process, and a higher liquid fraction can be achieved in this case. The numerical results show that the complete melting time is approximately 155 min for the pure PCM case, while the addition of 3% vol Al_2_O_3_ nanoparticles drives the complete melting time down to about 132 min. This means that the addition of 3% vol Al_2_O_3_ nanoparticles reduces the melting time of PCM by approximately 15%, which significantly improves the PCM melting performance in thermal energy storage systems.

### 3.4. Effect of Nanoparticle Material on PCM Melting Performance

As mentioned in Section 3.3, the PCM melting performance can be improved by adding nanoparticles due to their high thermal conductivity. The conventional idea is that the nanoparticles with higher thermal conductivity are expected to achieve better PCM melting performance. For example, according to the conventional theory, copper nanoparticles should show significantly better performance than Al_2_O_3_ nanoparticles because the thermal conductivity of copper is 10 times higher than that of Al_2_O_3_. In this section, we try to determine whether melting performance can be dramatically enhanced by using nanoparticles with much higher thermal conductivity.

**Figure 12:**
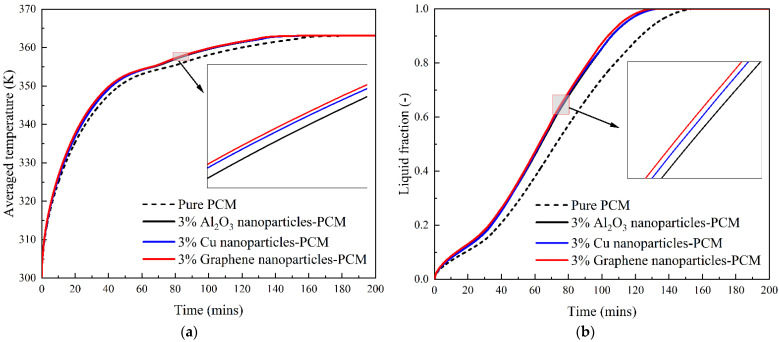
Effect of Al_2_O_3_, Cu, Graphene nanoparticles on the melting process of PCM. (**a**) Average temperature; (**b**) Liquid fraction.

The candidates chosen were Al_2_O_3_, copper and graphene nanoparticles; our aim was to understand their ability to enhance PCM melting performance. The thermal conductivity of these three nanoparticles varies from 36 to 5000, and their detailed thermophysical properties as listed in Table 2 in Section 2.3. Figure 12 depicts the distributions of average temperature and liquid fraction along with the PCM melting time for four different cases, including the pure PCM case, 3% vol Al_2_O_3_ nanoparticle-based PCM, 3% vol copper nanoparticle-based PCM and 3% vol graphene nanoparticle-based PCM. It can be seen that, compared to the pure PCM case, these three nanoparticles indeed enhance the PCM melting performance in a tubular energy storage unit. However, there is no noticeable difference among these three different nanoparticles in terms of improving the PCM melting process, although the enlarged figure demonstrates a very tiny difference among them, namely that performance increases slightly from Al_2_O_3_ to copper and graphene with increases in the thermal conductivity. This indicates that PCM melting performance cannot be remarkably improved even if we increase the thermal conductivity of nanoparticles from 36 to 5000. This is because the experimentally validated Formula (13) does not generate dramatic differences based on the thermal conductivity of Al_2_O_3_, copper and graphene nanoparticle-based PCM nanofluids, which are around 0.21824, 0.21853 and 0.21855, respectively. This suggests that, in addition to high thermal conductivity, we also need to consider other factors when choosing nanoparticles to enhance the thermal performance of PCM, i.e., their densities and costs. For instance, compared to Al_2_O_3_, copper’s higher density may render it unsuitable for applications of enhancing PCM performance in aerospace engineering, despite the fact that copper has a much higher thermal conductivity.

Equations (12)–(14) are employed to evaluate the effect of the Brownian motion of nanoparticles, which are used in the present numerical simulation. However, we need to keep in mind that Equation (12) and the empirical correlations for *β_k_* in Equation (13) are oriented for Al_2_O_3_ nanoparticles. As we did not find a suitable expression of the viscosity in Equation (12) or correlations for *β_k_* in Equation (13) for copper or graphene nanoparticles, the same expressions of Equations (12) and (13) are used for these two nanoparticle materials in our simulation. These two variables need to be further investigated by experimental studies in the future.

## 4. Conclusions

The role of Al_2_O_3_ nanoparticles in enhancing the performance of the PCM melting process was analysed in a tubular energy storage unit. Adding 3% vol Al_2_O_3_ nanoparticles into the PCM accelerated the melting process by 15%, which significantly improved PCM melting performance for thermal energy storage systems. We also analysed the mechanism by which nanoparticles with various thermal conductivities to improve the PCM melting performance, based on analyses of Al_2_O_3_-, copper- and graphene-based nanofluids with thermal conductivities ranging from 36 to 5000. We demonstrated that the graphene nanoparticles, with a thermal conductivity of 5000, do not significantly enhance the PCM melting process compared to the Al_2_O_3_ nanoparticles with a thermal conductivity of 36. This result occurred due to the fact that nanoparticles with higher thermal conductivities cannot significantly improve the thermal conductivity of PCM nanofluids according to the experimentally validated formula for the calculation of the nanofluids’ thermal conductivity, which are around 0.21824, 0.21853 and 0.21855, respectively, for Al_2_O_3_, copper, and graphene.

In addition to the nanoparticles’ enhancement of the performance of the PCM melting process, the addition of nanoparticles to the PCM system has some disadvantages, including a reduction in the thermal energy storage capacity due to the decrease in the overall volume of PCM [36,37]. In addition, the size represents a strong influence on nanoscale thermal effects [38]. In future studies, we would like to discuss whether fractional thermal transport can be useful for future research of candidate nanoparticles to be incorporated into phase change materials for thermal energy storage systems.

## Figures and Tables

**Table 1:** Thermophysical properties of the phase change material RT82 [25].

Thermophysical Properties	RT82
Solid density (kg m^−3^)	950
Liquid density (kg m^−3^)	770
Specific heat (J kg^−1^ K^−1^)	2000
Thermal conductivity (W m^−1^ K^−1^)	0.2
Latent heat (J/kg)	176,000
Dynamic viscosity (kg/m s)	0.03499
Solidus temperature (K)	350.15
Liquidus temperature (K)	358.15
Thermal expansion coefficient (1/K)	0.001

**Table 2:** Thermophysical properties of nanoparticles, including Al_2_O_3_, copper and graphene [26].

Thermophysical Properties	Al_2_O_3_	Copper	Graphene
Solid density (kg m^−3^)	3600	8920	2200
Specific heat (J kg^−1^ K^−1^)	765	380	790.1
Thermal conductivity (W m^−1^ K^−1^)	36	400	5000

## Data Availability

The research data supporting this publication are provided within this paper.
